# Lightweight Differentiated Transmission Based on Fuzzy and Random Modeling in Underwater Acoustic Sensor Networks

**DOI:** 10.3390/s23156733

**Published:** 2023-07-27

**Authors:** Jiabao Cao, Jinfeng Dou, Jilong Liu, Hongzhi Li, Hao Chen

**Affiliations:** 1 School of Science, Qingdao University of Technology, Qingdao 266520, China; caojiabao@qut.edu.cn (J.C.);; 2 College of Information Science and Engineering, Ocean University of China, Qingdao 266100, China

**Keywords:** underwater acoustic communication, directional transmission, lightweight load, performance optimization

## Abstract

Energy-efficient and reliable underwater acoustic communication attracts a lot of research due to special marine communication conditions with limited resources in underwater acoustic sensor networks (UASNs). In their final analysis, the existing studies focus on controlling redundant communication and route void that greatly influence UASNs’ comprehensive performances. Most of them consider directional or omnidirectional transmission for partial optimization aspects, which still have many extra data loads and performance losses. This paper analyzes the main issue sources causing redundant communication in UASNs, and proposes a lightweight differentiated transmission to suppress extra communication to the greatest extent as well as balance energy consumption. First, the layered model employs layer ID to limit the scale of the data packet header, which does not need depth or location information. Second, the layered model, fuzzy-based model, random modeling and directional-omnidirectional differentiated transmission mode comb out the forwarders step by step to decrease needless duplicated forwarding. Third, forwarders are decided by local computation in nodes, which avoids exchanging controlling information among nodes. Simulation results show that our method can efficiently reduce the network load and improve the performance in terms of energy consumption balance, network lifetime, data conflict and network congestion, and data packet delivery ratio.

## 1. Introduction

With the increasing intensity of marine resource development and the rapidly growing amount of marine data, underwater acoustic communication has attracted more and more attention in the underwater remote sensing field [1,2,3]. Underwater acoustic sensor networks (UASNs) are widely used in marine resource exploration, data collection, environmental monitoring and marine rescue [4,5,6]. However, UASNs deploy sensors with limited resources in the marine environment that are difficult to replace. When partial sensors run out of energy, UASNs do not work [7,8]. In the complex and varying ocean environment, underwater acoustic communication suitable for long-distance communication features long propagation delay, low bandwidth and a poor channel capability when the underwater speed of sound is about 1500 m/s, five times slower than that of radio waves (3 × 10^8^ m/s) [9]. Therefore, providing energy efficiency and reliable data transfer is the primary consideration for network performance in UASNs [10]. Most related studies come down to trying to decrease extra communication and route void, greatly influencing a UASN’s comprehensive performance. 

Redundant data transmissions increase the traffic pressure of UASNs, waste energy, and enhance the probability of data conflict and network congestion [11,12]. The traditional multi-hop routing protocols use unicast forwarding to choose single-route or multi-route forwarding, while they frequently exchange the collected information about the neighboring nodes to maintain the route [13,14]. Recently, opportunistic pouting (OR) uses broadcast forwarding by omnidirectional antennae to ensure network performance (such as the interconnection of communication, etc.), whereas multiple forwarding copies weaken the communication effect in the underwater acoustic channel and waste energy [15]. Some directional transmissions try to adjust the effective selection range of forwarders to control duplicated forwarding [10], while they might receive no successful forwarding ACK and cause multiple extra forwarding because coordination forwarders are not in the communication range of those with higher priority. And some location-based routing protocols and depth-based routing protocols select partial nodes toward the sinks or autonomous underwater vehicles (AUVs) as relay nodes under some routing metric, but these protocols need to transmit a longer data packet, including depth or location information, in the data forwarding process, or to interactively communicate some controlling information [14,16]. In addition, the uneven distribution of the load results in routing voids or long detours [17,18]. Therefore, extra data forwarding, redundant information exchange and uneven data load reduce the efficiency of acoustic communication and shorten the UASN lifetime [19]. 

**Figure 1:**
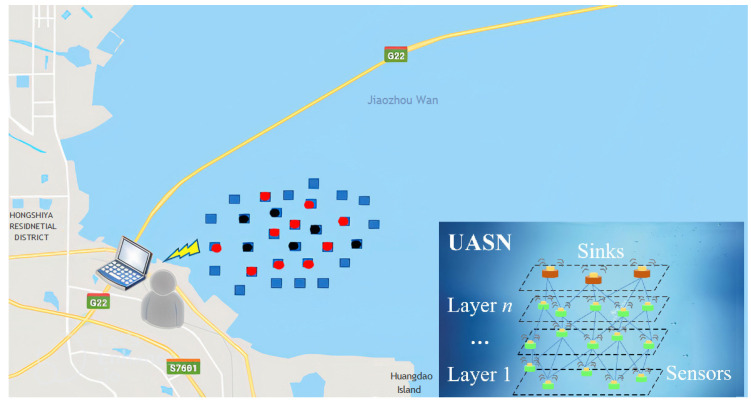
UASN remote sensing and ineffective network running example.

According to the existing protocols, Figure 1 shows the ineffective network running that occurs in the environmental monitoring of a coastal area, induced by a large number of excessive and uneven data loads. Since some black nodes are frequently chosen as relay nodes to forward the data, they consume too much energy, die prematurely and cause routing voids. Red nodes carry and exchange so much controlling information as to cause frequent data conflict and heavy network congestion. The multi-forwarding of data copies also aggravates the network overload and causes transmission delay. The phenomenon brings great difficulty to marine monitoring [20]. Because UASNs have a high deployment cost, limited bandwidth capacity, low energy efficiency, poor channel, etc., reducing redundant communication need to address the following issues: P1: A data packet contains many unnecessary controlling information. P2: Nodes need to exchange extra controlling information each other during each data transmission. P3: The broadcast feature of the omnidirectional or directional antenna enables the same data packet to be received by multiple nodes, which can lead to multiple forwarding copies. The existing protocols partially focus on P2 or P3. This paper addresses the above three issues and proposes a lightweight communication, called LLF-FR. In LLF-FR, we employ the reasonable allocation of the directional-omnidirectional hybrid transmission mode to reduce the redundancy communication as much as possible, which is different from the existing studies. The crucial contributions of our research are as follows.

- To solve P1, we build a layered network model. The data packets employ layer ID, instead of depth or location information, which reduces the length of the data packet header and data transmission load. In order to solve P2, the selection of relay nodes is carried out by local computation in nodes, avoiding the regular exchange of much of the controlling information among the nodes, such as location, residual energy, and so on.
- We design the layered concept to select initial candidate relays based on directional transmission, which limits the number of forwarders. Next, we propose the fuzzy model to further reduce the number of potential relay nodes as well as balance the energy consumption. Finally, the light-load efficient forwarding precisely chooses the optimum node to forward the data by random modeling. In the communication, we employ directional-omnidirectional differentiated transmission, which is different from single directional transmission where failed ACK receiving can cause some extra forwarding in the coordination process of forwarders. All these can effectively alleviate P3 and avoid multiple duplicated forwarding. Meanwhile, we consider the impact of marine acoustic velocity in the random model to coordinate the network delay.
- We perform extensive simulations to verify our method under multiple performance indicators. A large number of experimental results demonstrate that our method can better reduce network load, improve the energy efficiency, balance the energy consumption, prolong the lifetime of UASNs, improve the data packet delivery ratio, and reduce the probability of data conflict and network congestion.

The remainder of this paper is organized as follows. Section 2 presents the related work. The UASN network model, the energy consumption model and the ocean acoustic-velocity model are introduced in Section 3. We propose the data packet format optimization based on the layered model in Section 4. Section 5 describes the details of the proposed method choosing the optimum forwarder, including layer-based candidate relays, fuzzy object function modeling, random forwarding modeling and directional-omnidirectional differentiated transmission. In Section 6, the performance evaluation is discussed. Section 7 concludes this paper. The key parameters used in this article are listed in Table 1.

## 2. Related Work

In this section, we discuss the related communication protocols in USANs. They forward the data packets to the partial neighbor under some routing metric or node cooperation which can relieve P2 or P3 to some extent. 

(1) Location-based routing communication. The location-based routing protocols attempt to optimize P3. The vector-based forwarding protocol (VBF) [21] and VBF-based hop-by-hop protocol (HH-VBF) [22] are the first location-based opportunistic routing for UASNs and establish a “routing pipe” to transmit data packets to the destination. The location information of each node is used as the route metric to select the next hop forwarders. Only nodes inside the “routing pipe” join in forwarding, which tries to reduce forwarding duplication and data load. In [16], a typical geographic and opportunistic routing with depth adjustment (GEDAR) is proposed. The geographic information is required when deciding candidate forwarders, and the depth information is also required to avoid the route void by adjustment. The routing process wastes some energy due to lack of the energy factor. Aiming at achieving energy balance and reducing data conflicts, the energy-balanced VBF protocol [23] uses the residual energy as the expectation factor and adjusts the coordination time in each transmission round. In [24], a power control-based opportunistic routing (PCR) establishes a set of candidate forwarders under different transmission powers which minimizes energy consumption. 

In [8], an OR based on directional transmission (ORD) uses the coordination of neighbor nodes, the azimuth angle, the ratio of residual energy and the packet delivery ratio (PDR) as route metrics to calculate the forwarding utility of relay nodes, improving PDR, while it needs the location information and information exchange. Moreover, the directional transmission can cause multiple needless forwarding because coordination forwarders are not in the communication range of the best one and receive no forwarding ACK.

The location-based routing protocols have a tough assumption that each sensor node knows its own geographical information. They often exchange the location information among the sensors, which consume significant energy. In addition, obtaining the location information in the ocean remains another challenge.

(2) Depth-based Routing Communication. The depth-based routing protocols, partially P2 and P3. In depth-based routing communication, the nodes can obtain the depth by local pressure sensors. They are in an independent location, which avoids the regular exchange of location information [25]. 

Depth-based routing (DBR) [26] use single-metric depth difference to decide candidate relays, which greedily forwards data packets to the nodes with lower depth towards the surface sinks. DBR has no consideration for the priority rotation and balancing energy consumed among the nodes but attempts to minimize energy consumption, which causes a shorter network lifetime. In energy-efficient depth-based routing (EEDBR), the route metrics, depth and residual energy, are employed to determinate the forwarders [27]. A set of neighbor nodes with lower depth are selected as candidate forwarders, and then the nodes with high residual energy have short holding time and forward data packets. EEDBR introduces many additional information loads and is faced with a higher maintenance cost because each node need to maintain a neighbor table by periodically sending hello packets. 

Balanced energy consumption-based adaptive routing (BEAR) selects forwarders with relatively higher residual energy than those of the average network by mixed routing and cost functions by network sector division [28]. In each transmitting period of the forwarding process, the residual energy and identifying formation of nodes are required for exchange among the nodes for calculating the average remaining energy, which severely increases the network load, whereas the method of transferring the data to sinks either direct or via multi-hop can balance energy consumption. The energy balancing routing protocol (EnOR) [29] chooses the candidate forwarders according to the priority level decided by the remaining energy, link reliability and packet advancement. EnOR can balance the energy consumption of nodes, but it consumes a lot of energy because beacon packets are regularly broadcasted to obtain information from neighbor nodes in the routing process. In [30], the authors propose an opportunistic void avoidance routing protocol (OVAR) to choose the forwarders under packet delivery probability and depth. In order to avoid route voids, the hidden nodes in the forwarders are discarded by the building adjacency graph in each node. The energy and depth variance-based opportunistic void avoidance scheme (EDOVE) [31] selects the forwarding nodes with the maximum residual energy among their one-hop neighbors and more neighbors with a lower depth, to achieve energy balancing and void avoidance. To solve the route void and long detour, the distance-vector-based OR (DVOR) [18] introduces the distance vector by a query mechanism. It records the smallest hop counts toward the sinks, along which the data packet is forwarded hop by hop. It consumes more energy since the shortest hop cannot ensure the shortest transmission distance and it transmits query packets periodically. 

In the sparse UASN, a reinforcement learning-based opportunistic routing protocol is proposed to select the forwarding nodes set and cope with the routing void; the nodes in a forwarding set can monitor each other to suppress retransmission and transmission conflicts, reducing the probability of packet loss [32]. Q-learning-based opportunistic routing (QBOR) is studied under the neighbors’ residual energy and PDR [33]. Complex computing and information interact of nodes bring more cost to UASN with limited resources. An Objective Function (OF) is developed that uses fuzzy logic to dynamically adapt to variable environments in wireless networks, taking several metrics into account [34]. However, when multiple metrics are similar, the OF will obtain multiple forwarding nodes, which lead to more repeated forwarding.

As illustrated in Table 2, most communication protocols considered one or two issues still existing many redundant communications, and some protocols also pay no attention to balancing the energy consumption to decrease route void, while it proves our scheme reduces the extra data load to the most extent, and comprehensive optimizes the network performance by lightweight transmission. 

## 3. System Model

### 3.1. Network Model

**Figure 2:**
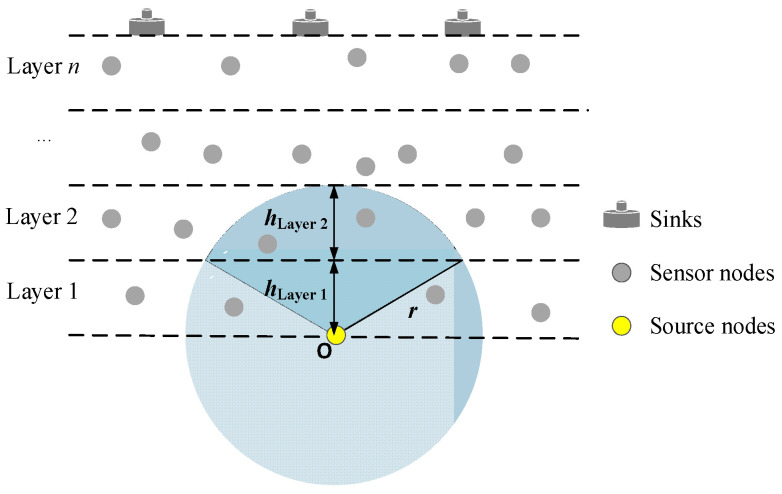
UASN Layered model. Sensor nodes with different depths are divided into Layer 1, Layer 2, …, Layer *n* from the bottom to the top, which sense and send the data toward the sink by directional transmission or broadcast. The upper valid nodes forward the data.

We consider a UASN layered model, as shown in Figure 2. The sensor nodes are randomly anchored to the seafloor or floated in the sensing sea area with different depth, and divided into Layer 1, Layer 2, …, Layer *n* from the bottom to the top according to the depth. The sensor nodes can sense data from the surrounding marine environment and send the data toward the sink by directional transmission or broadcast, its upper valid nodes meeting with forwarding condition forward the data. The sinks are distributed on the sea surface, and can communicate with the sensor nodes by acoustic communication links. Because the propagation loss of acoustic channels increases with the increase in both distance and signal frequency [35], multi-hop transmission is an ideal data transmission mode for long-distance acoustic communication in this paper. The sinks collect the data from sensor nodes, and then transmit them to the edge or cloud sever. 

We make some reasonable assumptions about the network model:

(1) Each sensor node can control its transmission power, and own computing power to support different MAC protocols and perform signal processing functions.

(2) Each sensor node has a unique identification and Layer ID, and gets its depth information via its own pressure sensor. 

(3) The monitoring energy consumption is much less than the communication energy consumption [36], so the monitoring energy consumption is not considered.

(4) The current directional underwater acoustic transducers and omnidirectional antenna can ensure the transmission [37]. 

### 3.2. Energy Consumption Model

Sozer et al. proposed the energy consumption model [36], quantifying energy consumption based on underwater acoustic communication. According to this model, the transmission power of each sensor node over a distance *d* is calculated as

(1) $$P_{t}=P_{0}A\left(d\right),$$

where *P*_0_ is the minimum receiving power of nodes, *A*(*d*) is the path loss [35] over the distance *d* for a signal of frequency *f*; it can be expressed as

(2) $$A\left(d\right)=d^{k}\alpha^{d},\;\alpha =10^{\alpha \left(f\right)/10},$$

where *k* is the energy spreading factor, with *k* = 1 for cylindrical propagation models, *k* = 2 denoting spherical propagation models, and *k* = 1.5 for practical propagation models. And α(f) is a frequency-dependent term obtained from the absorption coefficient. According to Thorp’s formula [35], the absorption coefficient in the underwater environment can be expressed as

(3) $$\alpha \left(f\right)=\frac{0.11\times f^{2}}{1+f^{2}}+\frac{44\times f^{2}}{4100+f}+2.75\times 10^{-4}f^{2}+0.003,$$

in dB/km for *f* in kHz.

Then, the energy consumption for a sensor node transmit *l* bit data over distance *d* can be expressed as

(4) $${\;E}_{T}\left(l,d\right)=P_{0}A(d)l/\varepsilon .$$

where *ε* denotes the transmission rate of node, and l/ε is the transmission duration.

The energy consumption for a sensor node receiving a packet of length *l* can be expressed as

(5) $$E_{R}\left(l\right)=P_{r}l/\varepsilon ,$$

where *P_r_* is receiving power which depends on the receiving devices.

As a result, the energy consumption is related to the transmission distance and the amount of transmission data. Decreasing the amount of redundancy data can save the energy.

### 3.3. Ocean Acoustic Velocity Model

We can use the Mackenzie nine-term equation to calculate *C* at different depths [38]:

(6) $$C=1448.96+4.591T+2.374\times 10^{-4}T^{3}-5.304\times {10^{-2}T}^{2}+1.340\left(S-35\right)\;-1.025\times 10^{-2}T\left(S-35\right)+1.675\times 10^{-7}D^{2}-7.139\times 10^{-13}TD^{3},$$

where *T* is the temperature in °C, *S* is the salinity in ppt, and *D* is depth in m. The applicable range of (6) is 0 ≤ *T* ≤ 35 °C, 30 ppt ≤ *S* ≤ 40 ppt, and 0 ≤ *D* ≤ 8000 m. We can see that the ocean acoustic velocity is related with the complicated ocean environment facts including temperature, salinity, depth, pressure, and so on. The ocean acoustic velocity *C* fluctuates according to various depths. As we know, *C* decreases monotonically with depth in the shallow-sea channels, because the upper ocean is strongly illuminated by the sun. When the depth is greater than 1 km in the deep-sea channels, *C* increases linearly with depth. At this time, the variance of static pressure almost becomes the only reason for the varying of *C* with depth [20]. Under the character of *C*, accordingly, the speed and time of the data transmission also varies with various ocean depths. We can coordinate the delay time with various ocean depths using *C*.

## 4. Data Packet Optimization Based on Layered Model

**Figure 3:**
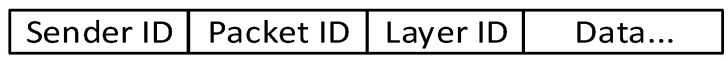
Packet format.

The layered network is used to forward the data packets. In the data packet, the layer ID can assist the forwarding process, which does not need to carry the information of the location or depth anymore. For the purpose of reducing the overhead of the header, the format of the data packet is improved as shown in Figure 3. Sender ID represents the source node ID, Packet ID indicates the serial number of the packet, Layer ID is the order number of the layer about the sending or forwarding node. Sender ID and Packet ID ensure the uniqueness of the data packet. Layer ID is updated hop-by-hop (layer-by-layer). Other fields remain unchanged during the forwarding process. This format reduces the transmission of extra control information. Furthermore, compared with the depth information, the Layer ID occupies fewer bits, and the length of the data packet is shorter. All of these can relieve the problem P1.

**Figure 4:**
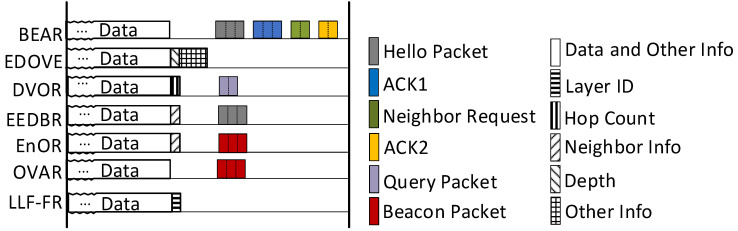
Control information transmitted for each protocol.

Figure 4 compares various control information that the several related routing protocols need to exchange during the communication process. It shows that the types, amount and scale of control information required by the LLF-FR are less than those of the other protocols. The black and white entries indicate various control information that needs to be carried in the header of the data packet, and the colored entries indicate various control packets that the node needs to transmit periodically. It can be clearly seen that compared with other protocols, the LLF-FR proposed in this paper requires very few types of control information (to solve P1), and does not need to exchange control packets (to solve P3), which greatly reduces the amount of information transmitted in the network. It lightens the communication load and the network energy consumption. 

**Figure 5:**
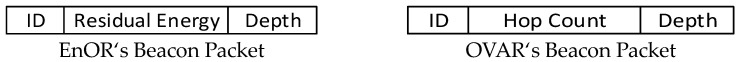
Sample of control package.

In different protocols, some control packages with the same name may have different types of control information, but the number of types is the same. For example, both EnOR and OVAR have control packets named as Beacon packets, but they contain different control information, as shown in Figure 5.

## 5. Optimum Forwarder Decision with Directional-Omnidirectional Hybrid Mode

LLF-FR further designs the lightweight forwarder scheme to retrench the number of the forwarding node, as well as to balance the energy consumption, including the initial candidate relay node set based on the transmission layered model, the potential forwarding nodes based on the fussy model, the efficient forwarding scheme based on random modeling and the sensors’ directional-omnidirectional hybrid transmission mode. These steps precisely choose the optimum node to forward the data, can effectively cope with P3 and avoid multi-forwarding duplicates. Furthermore, the selection of the relay node set is carried out by local computation in the nodes; neither needs to regularly communicate controlling information to each other, nor do they need to carry the forwarders information in the packet header. Thus, they can also avoid P3. Consequently, the scheme can save energy, balance energy consumption, reduce the risk of data congestion and data conflicts, improve the delivery ratio of data packets and effectively use bandwidth, by the reasonable transmission reduction of control information, extra data copies, and multi-forwarding duplicates. 

### 5.1. Candidate Relay Nodes Based on Layered Transmission

The layered model selects the nodes within the sender’s directional transmission range whose layer IDs are larger than those of the sending nodes, as candidate relay nodes, and, accordingly, pruning the range of forwarding nodes. The set of candidate relay nodes, $F_{c}\left(i\right)$, is expressed as

(7) $$F_{c}\left(i\right)=\{Node\;j\in N|\left({Layer}_{Node\;j}>{Layer}_{Node\;i}\right)\cap (d_{ij}\leq r)\},$$

where *N* represents the neighbor nodes of the sending node, and Layer_Node *j*_ and Layer_Node *i*_ represent the layer ID of the receiving node and the sending node, respectively. *d_ij_* is the distance between the receiving node *j* and the sending node *i*, *r* is the transmission range of sending node *i*. The calculation of *d_ij_* is unnecessary because of the broadcast character of the sending node. Every Node *j* that can receive the data packet from Node *i* satisfies the relation, $d_{ij}\leq r$. The $F_{c}\left(i\right)$ of the sending nodes decides if a node is a qualified candidate relay node. When Node $\;j\in F_{c}\left(i\right)$, it is a qualified candidate relay node. If Node $j\notin F_{c}\left(i\right)$, it will discard the received packets. 

Here, to ensure that the nodes located near the boundary of each layer can achieve cross-layer communication, we define the reference relation between the node transmission radius *r* and the layer height as

(8) $$r=h_{Layer\;i}+h_{Layer\;i+1},$$

where *h*_Layer *i*_ is the height of Layer *i*, and *h*_Layer *i*+1_ is the height of Layer *i* + 1. Figure 2 shows an example of the boundary Node *O*. 

In addition, according to assumption 1, the transmission range of the node can be adjusted by the power control. In this way, if the nodes encounter the void region, the transmission range can be expanded appropriately to explore the forwarding nodes. We can evaluate the number of candidate forwarding nodes for each node. We set the node density of UASN as $\rho_{A}$, and the volume of the upper candidate region within the directional transmission range of the sending node as *V_i_*. Then, the number of candidate relays of sending nodes can be obtained as $N_{i}=\rho_{A}V_{i}$. Therefore, we can set suitable $\rho_{A}$ to adjust the number of candidate relays, and adjust the node power to successfully find the forwarder. The forwarding of data packets will fail only if all nodes in the forwarding set are unable to transmit the data packets.

### 5.2. Fuzzy-Modeling-Based Potential Relay Nodes

It is necessary for designing the optimum forwarding plan to refer to many performance aspects such as the load, energy, energy consumption balance, delay time, etc. and the corresponding metrics. LLF-FR introduces the fuzzy model that can set many metrics to set the priority level of the candidate relay nodes according to the real requirements. When the data arrive at the candidate relay nodes of $F_{c}\left(i\right)$, the nodes with highest priority are chosen as the potential relay nodes $F_{p}\left(i\right)$. Each node has many linearly independent metrics. We define the metrics set of Node *j* ${\in F}_{c}\left(i\right)$ as the linear matrix $M_{j}$ and the normalized vectors matrix as H. Then, the priority of candidate relay node *j* is calculated by

(9) $${Priority}_{j}=\left(\alpha_{0},\alpha_{1},\ldots ,\alpha_{k}\right)\cdot M_{j}\cdot H^{-1}=\left(\alpha_{0},\alpha_{1},\ldots ,\alpha_{k}\right)\left[\begin{array}{cccc} m_{0} & & & \\ & m_{1} & & \\ & & \ddots & \\ & & & m_{k} \end{array}\right]{\left[\begin{array}{cccc} \gamma_{0} & & & \\ & \gamma_{1} & & \\ & & \ddots & \\ & & & \;\gamma_{k} \end{array}\right]}^{-1},$$

where *m_i_* (*i* = 0, 1, …, *k*) represents each available metric for Node *j*, and *k* is the number of available metrics. *γ_i_* is the normalized vector corresponding to each metric. We set one normalization vector, because each metric has the same value range no matter which node. *α_i_* (*i* = 0, 1, …, *k*) represents the coefficient corresponding to each metric *m_i_*, which is adaptive and can be adjusted according to the real application. 

The metrics in the fuzzy model can be set according to each real case and can satisfy the overall performance requirement. Here, we made a detailed design. In order to balance the energy consumption of each node, we need to set the remaining energy as one metric. Because each node’s energy is almost the same at the beginning, if only using the remaining energy as one metric to calculate the priority, each node will have the same priority and forward the data copies at the same time. We considered the depth as another metric and chose the relay node according to the depth, which can avoid too many nodes in $F_{c}\left(i\right)$ simultaneously forwarding a lot of duplicated data, although this method cannot even out the energy consumption. Therefore, we chose *E_o_* − *E_r_*, the difference between the initial energy *E_o_* and the residual energy *E_r_*, as *m*_0_ and chose the depth metric as *m*_1_. The corresponding energy normalized vectors were defined *γ*_0_ = *E_o_*, and *γ*_1_ is the depth of the deepest node in Layer 1. The coefficient (*α*_0_, *α*_1_) is defined as

(10) $$\left(\alpha_{0},\alpha_{1}\right)=\left\{\begin{array}{c} \left(+\infty ,+\infty \right)\;\;\;\;\;if\;E_{r}<E_{low}\\ \left(1,\;0\right)\;\;\;if\;E_{low}\leq E_{r}\leq E_{o}-\\ \left(0,\;1\right)\;\;\;\;\;\;if\;{\;E}_{0}-\lambda <E_{r} \end{array}\right.\lambda ,$$

where the residual energy *E_r_* = *E_o_* − *m*_0_, *E_low_* is the lowest node energy limitation to transmit data packet, *E_o_* is the initial node energy and *λ* is the threshold. When the node energy is below *E_low_*, it can hardly send data packets anymore. When the energy of each node is almost equal to *E_o_*, the depth metric will be main factor to select the forwarding node. In the other condition, the node with the biggest *E_r_* will forward the data in turns. Thus, we could take turns to choose the candidate relay nodes according to their priority to balance the energy consumption. And we calculated the Priority*_j_* of each Node *j* ${\in F}_{c}\left(i\right)$. Finally, we can obtain the fuzzy-based objective function:

(11) $$O_{j}=\underset{\mathrm{Node j}\;\in \;F_{c}\left(i\right)}{\min}({Priority}_{j}),$$

where $F_{c}\left(i\right)$ is the candidate relay nodes set of sending node *i*. According to the above objective function formula, the nodes with the lowest score of Priority*_j_* are further chosen as the potential relay nodes set, $F_{p}\left(i\right)$.

Algorithm 1 shows the implementation process of $F_{c}\left(i\right)$, Priority*_j_*, *Oj*, $F_{p}\left(i\right)$.
**Algorithm 1** $F_{c}\left(i\right)$, Priority_*j*_, $F_{p}\left(i\right)$Input: sensor nodes, sinks, *E_o_*, *E_r_*, Layer ID, Depth, *E_low_*, *λ*, *γ*_1_ Output: $F_{c}\left(i\right)$, Priority*_j_*, $F_{p}\left(i\right)$ 1. **while** Node *j* receives the packet *P_i_* **do** 2.      Obtain the Layer ID of *P_i_*; 3.     **if** Layer ID of Node *j* > Layer ID of *P_i_*
**then** 4.       Put Node *j* into $F_{c}\left(i\right)$; 5.    **endif** 6.    return 7. **endwhile** 8. **for**
∀ Node *j*
${\in F}_{c}\left(i\right)$ 9.   Compute Priority*_j_*, *O_j_* by (9)–(11); 10: **endfor** 11: Choose nodes with lowest *O_j_* into $F_{p}\left(i\right)$


### 5.3. Random Modeling

We can obtain fewer forwarding nodes from the potential relay node set $F_{p}\left(i\right)$ by the fuzzy model function *O_j_*. However, when *E_o_* and the depth of several nodes are similar, they will have the same score of Priority*_j_*, and forward the data packets repeatedly, which can also result in redundant transmission. Hence, LLF-FR proposes a random modeling to enhance the priority and coordination of the potential relay nodes. When the data packet arrives at the nodes in $F_{p}\left(i\right)$, the nodes keep the data packets for short coordination holding (*CoH*) time. The node with shorter *CoH* time will participate in forwarding. If a node forwards the data packet successfully, the other nodes in potential relays will receive the information of the data packet successfully forwarded. Thus, they know the transmission of that packet by a node with a shorter *CoH* time, then stop the forwarding schedule and discard the data copies. 

**Figure 6:**
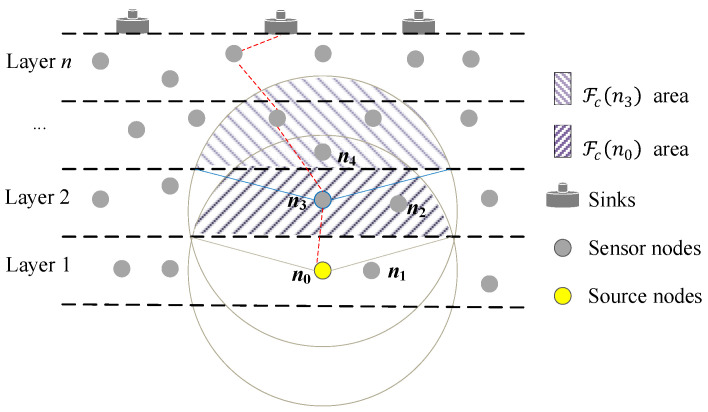
Example of effective relay nodes and directional-omnidirectional transmission.

For example, in Figure 6, nodes *n*_2_, *n*_3_, *n*_4_ are in the $F_{p}\left(n_{0}\right)$, and nodes *n*_2_ and *n*_4_ are in the transmission range of node *n*_3._ When node *n*_3_ with the shortest *CoH* time successfully forwards the data packet from node *n*_0_, nodes *n*_2_ and *n*_4_ will discard the same data packet from node *n*_0_ and stop forwarding its duplicated packet. This way further reduces the data load and the probability of packet collisions. We defined the *CoH* time of Node *j* as

(12) $$CoH_{j}=\mu {Priority}_{j}+\eta \frac{r}{C}+\beta \cdot {rand}_{j}\left(0.1,\sigma \right)\cdot \frac{r}{\bar{c}},$$

where *μ*, *η*, *β* indicate the adjustment coefficient, which can be set according to the real situation, and *r* represents the transmission range of the node. The acoustic velocity *C* is from (6), and $\bar{C}$ is the average sound propagation speed in the ocean (usually set to 1500 m/s). The LLF-FR model introduces *C* varying with the different ocean depth to the *CoH* time model, aiming at coordinating the network time-delay. The model adopts a random number *rand* (0.1, *σ*) to appropriately adjust the *CoH* time difference of nodes where *σ* is a predefined value range and can be defined according to the requirements of the application environment. The random number can prevent other low-priority potential nodes from repeated forwarding if they do not receive the forwarding information from higher-priority node in time. The *CoH* time difference of any two potential nodes must be more than *r*/$\bar{c}$ in order to ensure that the low-*CoH* time nodes in the same potential relays set can hear the forwarding of high-*CoH* time nodes and give up the extra forwarding, when a node with shortest *CoH* time forwards the data packets successfully. Our LLF-FR can ensure this condition, the *CoH* time difference of any two nodes in $F_{p}\left(i\right)$ is more than *r*/$\bar{c}$. Appendix A gives the related theorem and proof. Therefore, this way can try to avoid extra forwarding duplicates both from the potential relay nodes in $F_{p}\left(i\right)$ with the same Priority*_j_* and from multiple nodes having similar *CoH* time, further cut down extra multi-forwarding.

Here, the values of *β* and the difference $\left|{rand}_{j}\left(0.1,\sigma \right)-{rand}_{k}\left(0.1,\sigma \right)\right|$ of any two nodes influence the *CoH* time. When σ is smaller, $\left|{rand}_{j}\left(0.1,\sigma \right)-{rand}_{k}\left(0.1,\sigma \right)\right|$ is also smaller; accordingly, the *CoH* time and network delay are reduced. There may exist more nodes with the same *CoH_j_* repeating the forwarding when σ is smaller. However, the $F_{p}\left(i\right)$ obtained by the fuzzy model ensures fewer potential forwarding, which can make smaller σ possible and avoid too much network delay. If the real communication has higher requirements for time delay, the value of *β* will be adjusted lower; meanwhile, several forwarding nodes will have similar delay time, forward the data packet at the same time, and cause few extra data packets. If the real communication has higher requirements on channel utilization, the value of *β* will be larger. At this time, the small time delay will be sacrificed, but the number of multiple forwarding nodes can be reduced as much as possible. This avoids the forwarding of many extra data packets and improve the channel utilization rate.

### 5.4. Directional-Omnidirectional Hybrid Mode

According to the layered forwarding model, Layer 1 is the lowest layer. Therefore, the nodes of Layer 1 only send the data packets generated by themselves and they do not need to play the role of the relay nodes. However, the nodes in other layers not only send their own data packets, but also forward the data packets from lower layer nodes. To refine the forwarder number and reduce the broadcast storm, the source sensors generating and sending the data packet employ directional transmission (e.g., *n*_0_ in Figure 2). In the process of forwarder collaborative competition, only the sensors (e.g., *n*_2_ and *n*_4_ in Figure 6) in the transmission range of the sensor (e.g., *n*_3_ in Figure 6) successfully forwarding the data packet can receive the information and stop its own forwarding schedule. If the successful forwarder uses directional transmission (e.g., *n*_3_ in Figure 6), some sensors cannot receive the information successfully forwarding (e.g., *n*_2_ in Figure 6), and then continue to forward the needless data copies. Considering the stripped-down number of candidate relay sensors decrease the extent of broadcast storm, we set the omnidirectional transmission for the forwarders to avoid the above case.

So, the source sensor nodes and the forwarding sensor nodes of upper layer (Layer *i* (*i ≥* 2)) run different algorithms. The running of the source sensor nodes is illustrated in Algorithm 2. Algorithm 3 summarizes the transmission details of forwarding sensor nodes. For preventing the packets from the broadcast storm, we set the AP buffer and CP buffer for each node. The AP buffer of the node records the packet ID of the packet that has already been forwarded, and the CP buffer temporarily records the current packets waiting for forwarding. When each node finds that its receiving packet ID has existed in the AP buffer, it indicates that that packet has been forwarded and the node will drop the packet. If the receiving packet has existed in CP buffer, it indicates that the current node has known that the receiving packet has been forwarded by a potential forwarder with the shortest *CoH* time. Therefore, the packet will be discarded and the Packet ID will be added to the AP buffer. If neither of these conditions are met, the receiving packet will add to the CP buffer, update the Layer ID of the packet header and start a *CoH* timer according to (12). Once the shortest *CoH* timer ends, the packet in CP buffer will be forwarded immediately.
**Algorithm 2** Data Transmission of Source Sensor NodesInput: *E_o_*, *r*, Layer ID, Depth Output: action of sensor node 1. **while** sensor node in Layer *i* generates Packet *P_i_* **do** 2.    Broadcasts Packet *P_i_* by directional transmission mode 3. **endwhile**

**Algorithm 3** Data Transmission of Forwarding Nodes Input: *E_o_*, *r*, Layer ID, Depth, *μ*, *η*, *β*, *C*, $\bar{C}$, Priority*_j_* Output: action of sensor node 1. **while** sensor Node *j* in Layer *i* receives Packet *P_i_* **do** 2.    **if** Layer ID of Node *j* = 1 **then** 3.      Discards Packet *P_j_*
4.       **else**
5.     Obtain Packet ID, Layer ID of *P_i_*; 6.      **if** Node $j\in F_{c}\left(i\right)$, **then** 7.       Look up AP buffer; 8.        **if** Packet ID of *P_i_* is in AP buffer **then** 9.         Discard Packet *P_i_*; 10.         **else** 11.           Update Layer ID in *P_i_*; 12.            Calculate *CoH_j_* by (12); 13.            Look up CP buffer; 14.            **if** Packet ID of *P_i_* is in CP buffer **then**
15.               Discard Packet *P_i_* and remove *P_i_* ID from CP buffer; 16.            **else**
17.               Add Packet *P_i_* into CP buffer; 18.            **endif** 19.               Forward Packet *P_i_* after *CoH_j_* ends by onmidirectional mode; 20.           **endif** 21.       **else if**
$Node\;j\notin F_{c}\left(i\right)$ **then**
22.           Look up CP buffer; 23.           **if** P*_i_* is in CP buffer, **then** 24.             Discard Packet *P_i_* and remove *P_i_* ID from CP buffer; 25.           **else** 26.             Discard Packet *P_i_*; 27.           **endif** 28.       **endif** 29.     **endif** 30. **endwhile**

## 6. Simulation Results

### 6.1. Parameter Setting and Related Definition

This part evaluates the performance of the proposed LLF-FR scheme. We randomly deployed sensor nodes in a cube monitoring region with a length of 1000 m. Several sinks are placed at the region top. We divided the monitoring area into four layers, and the average height of a layer was 250 m. Meanwhile, the LLF-FR was compared with the classical DBR [26], ORD [10], DVOR [18] and Flooding broadcast under the same experimental conditions. For fairness, the main parameters of LLF-FR (including omnidirectional and directional transmission powers, receiving power, data rate, etc.) were set referring to [10,18,26], as shown in Table 3. *E_low_* is the minimum energy required to forward a packet to the node. We set λ as 1% of initial energy. *γ*_0_ and *γ*_1_ were set according to the value range of the node’s initial energy and the depth of the deepest node in Layer 1, respectively. The simulation makes some definitions as follows:

(1) The network lifetime is defined as running rounds when the nodes with a given proportion die;

(2) The network load is defined as the total number of packets communicated in the network during each period; 

(3) The data delivery rate is defined as the ratio of the number of valid packets received by sinks to the number of packets actually sent by each node in the network;

(4) Each sensor node evenly senses and generates the data packet in each round.

In the simulation, we analyzed the set of the node density in order to decrease the void region. If we deployed 60 nodes in a fixed monitoring area, the volume of which is $1\times \sqrt{3}\times \sqrt{3}\;$= 3 km^3^, then the node density would be $\rho_{A}=20$/km^3^. According to Figure 2, the number of candidate relay nodes in the upper region within the transmission range of the sending node *O* was more than $\rho_{A}\int_{\;0}^{2\pi }d\vartheta \int_{0}^{\frac{\pi }{3}}d\varphi \int_{0}^{\frac{1}{4}}r^{2}\sin\varphi dr>2$. Therefore, we can reasonably deploy the node density and ensure that the nodes can find the effective forwarding nodes.

### 6.2. Simulation Comparison

**Figure 7:**
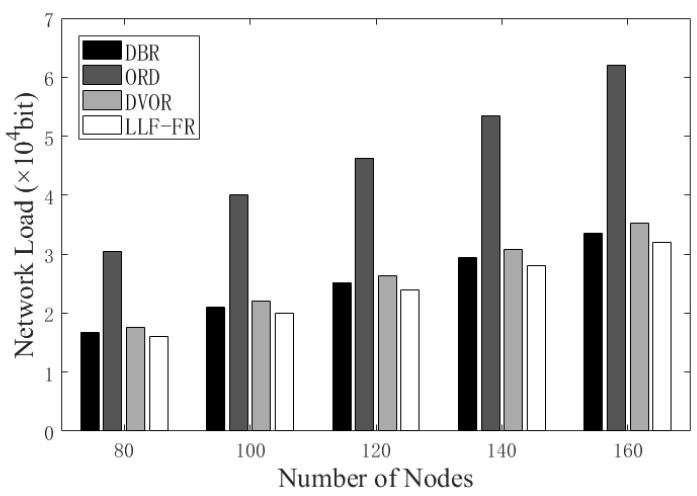
Network load with various numbers of nodes in the network.

Figure 7 simulates the network load with different node numbers in the network. LLF-FR has a lower network load than others, because LLF-FR cuts down the redundant communication as much as possible. DBR and DVOR have a higher load than LLF-FR, though the redundant packet control mechanism can reduce the network load to some extent. ORD is the worst because the exchange of control information (for example, location and ACKs) in the network causes redundancy load and a large network load, although it employs directional transmission to control the forwarder number. Moreover, in the coordination of forwarders, directional transmission maybe omits to inform some candidate forwarders so that they still forward extra data copies. LLF-FR employs the reasonable allocation of directional-omnidirectional transmission to avoid the problem and obtains better effects. 

**Figure 8:**
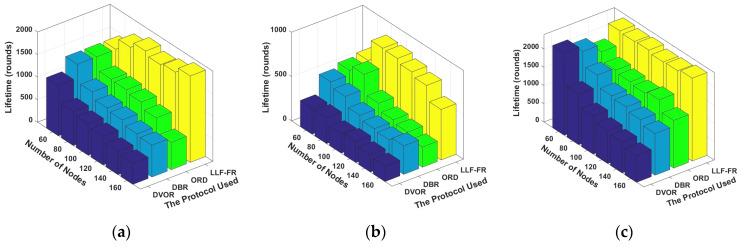
Network lifetime when (**a**) the first sensor node, (**b**) 30% nodes, and (**c**) 50% nodes exhaust their energy.

Figure 8a–c show the network lifetime when the first sensor node, 30% nodes or 50% nodes exhaust their energy, respectively. LLF-FR outperforms others due to less data load and a more balanced energy consumption. DVOR uses hop count as a metric to select forwarding nodes, without consideration of the energy consumption, which results in a short network lifetime. DBR chooses the relay nodes only based on the depth information; both optimizing and balancing energy consumption are not considered. Hence, the lifetime is worse. As DBR reduces the duplicate forwarding packets, it is better than DVOR. The ORD exchanges a large amount of control information and consumes a lot of energy. However, the ORD considers directional transmission and reduces forwarders, so it has slightly longer network lifetime than DVOR and DBR. 

**Figure 9:**
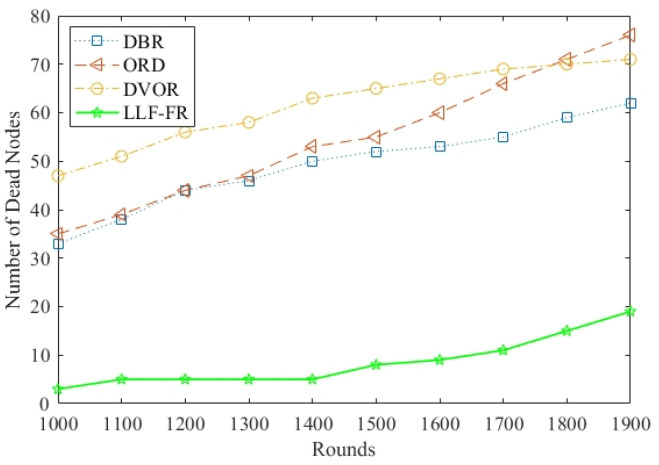
Number of dead nodes in different rounds.

In UASNs, the death of partial nodes may lead to routing void holes in local areas, affecting data monitoring and transmission; therefore, Figure 9 compares the number of dead nodes in the different running rounds where there are 100 nodes in the network. The number of dead nodes in LLF-FR is the lowest with various rounds, since LLF-FR uses the priority rotation of each forwarder to balance the energy consumption of nodes. Furthermore, lightweight load of LLF-FR saves node energy which prevents the nodes from exhausting energy prematurely. DVOR and DBR do not consider node energy optimization, and their use of hop count and depth do not change over time. Therefore, nodes with fewer hops and shallower depths are always selected, resulting in faster deaths. Moreover, DVOR uses an additional beacon mechanism, which requires regularly obtaining hop information from each node through beacon packets, resulting in additional energy consumption. So, DVOR generates more dead nodes. In ORD, nodes always die so fast because of its extensive exchanges of control packets. 

**Figure 10:**
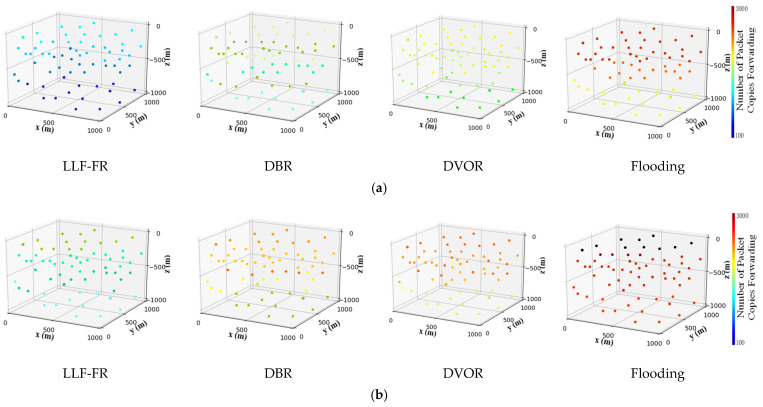

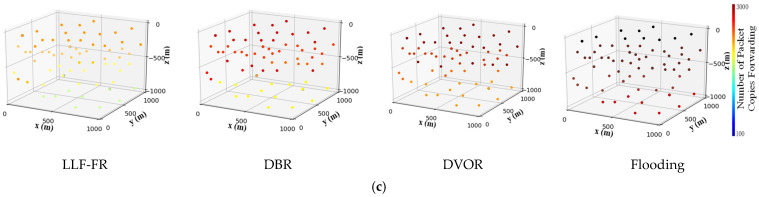
(**a**) Total number of times each node forwarding packet running until the 100th round. (**b**) Total number of times each node forwarding packet running until the 300th round. (**c**) Total number of times each node forwarding packet running until the 500th round.

For comparing the probability of data conflict and network congestion in the different protocols, Figure 10a–c shows the total forwarding frequency of the packet copies in each node when LLF-FR, DBR, DVOR and Flooding run to different rounds, respectively. The color card on the right of the figures defines the forwarding frequency and color sequence. The color transition varies from blue to red with the order from bottom to top. The color will gradually change from blue to red when the times of the node forwarding packets increase. Starting from the initial running of the network, each time the node forwards one packet, it is dyed in the figure according to the color sequence in the color card. With the increase in the packet forwarding times, the color is deepened according to the color sequence from bottom to top. When the forwarding times grows continuously, the color of the nodes become deeper and deeper. As we can see from the figure, the color of each node in LLF-FR can remain relatively light and even under different rounds. The results indicate that each node has a lower frequency of forwarding data, and the nodes within the same layer have a relatively balanced forwarding frequency in LLF-FR, which is better than the other two protocols. So LLF-FR can better reduce the probability of data conflict and network congestion. DBR only considers depth as the condition for choosing forwarding nodes, and the forwarding frequency of nodes is not balanced; therefore, the color of nodes is darker and unbalanced, as shown in Figure 10. DVOR uses hop count as a measure to select forwarding nodes, but nodes have to update hop count to sink periodically. Therefore, more packet copies are delivered in the network, resulting in a higher forwarding frequency of nodes and thicker color compared with DBR and LLF-FR. The Flooding protocol does not limit the number of forwarding nodes, so more packet copies are transferred in the network, which results in higher forwarding frequency for each node. Consequently, the color of nodes in the Flooding protocol is very dark on the whole, with the poorest performance. 

**Figure 11:**
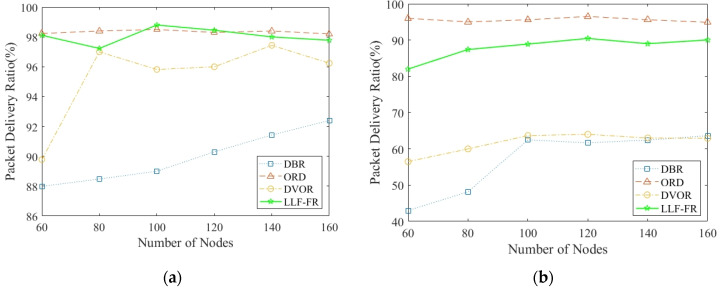
Packet delivery ratio varies with various number of nodes in the network: (**a**) when 30% nodes are dead; and (**b**) when 50% nodes are dead.

In order to verify the transmission reliability, Figure 11a,b compares the PDR of LLF-FR, ORD, DBR and DVOR when deploying different numbers of nodes, with the proportion of dead nodes being 30%, and 50%, respectively. The PDR using LLF-FR is higher than that of DBR and DVOR, and slightly lower than that of ORD. The reason is that ORD adopts the PDR metric to select the forwarders to improve PDR. But the cost of single directional transmission leading to some extra forwarding and exchanging information can increase network traffic and consume more energy. LLF-FR reduces redundant data transmission, avoiding network congestion and packet loss caused by excessive data in the network. At the same time, its speed of generating dead nodes is much slower than other protocols, decreasing the impact of routing holes on the PDR. Overall, LLF-FR balances the relationship between energy consumption and PDR. As we can see in Figure 11b, due to the large number of dead nodes in the network, the PDR of each protocol decreased. ORD sacrificing energy and its PDR is a little higher; however, the UASN might already not work due to the earlier death of some nodes at that time.

**Figure 12:**
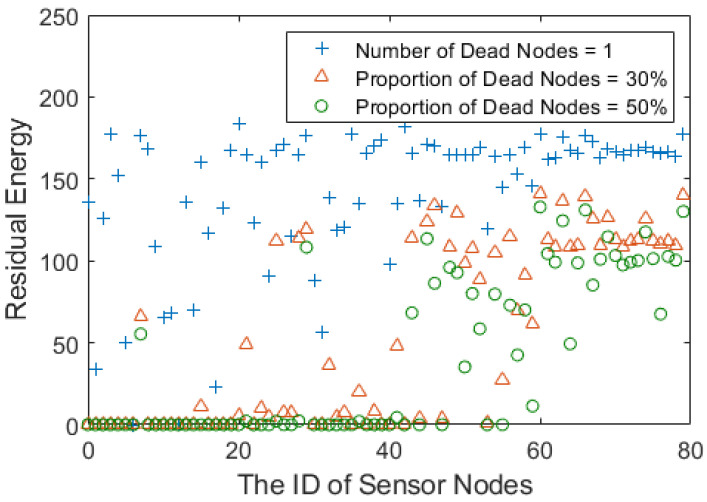
Residual energy distribution of nodes with different numbers of death nodes.

Figure 12 evaluates the energy efficiency and the uniformity of nodes’ energy consumption in LLF-FR by the residual energy of the nodes when the first node, 30% of nodes and 50% of nodes in the network run out energy, respectively. As shown in Figure 12, the local remaining energy is similar and balanced, and the remaining energy of the nodes in the lower layer is higher than that of the nodes in the upper layer at the same time. The reason is that the optimum forwarding node is chosen in the local layer in terms of the fairness of the local layer. Therefore, we are going to consider the heterogeneous network according to the network communication load or other methods in order to further balance the global energy and solve the global uniformity of energy consumed in the network.

## 7. Conclusions

This paper analyzed the characteristics of underwater acoustic communication and the focus of existing mainstream studies, and summarized three sources of redundant communication impacting the UASN comprehensive network performances, which presents a basis for studying better data-transmission protocols. And then LLF-FR optimization of the network overhead was designed by solving the three sources to suppress the extra data load. LLF-FR optimized the header controlling information and the length of the data packet, based on the layered network model, to refine the data load in the data transmission. The number of forwarders was cut down step-by-step by the candidate relay-nodes set, the fuzzy model, and the random model. Different from the single transmission mode, the reasonable allocation of the directional-omnidirectional transmission mode decides the optimum forwarding node with which to transfer data packet and efficiently limits the forwarding copies. Meanwhile, the defined forwarding models can take turns to choose the forwarder, which ensures the balancing load and an even energy consumption. The time delay was also coordinated considering the impact of the marine acoustic velocity in the random modeling. In addition, the selection of forwarding nodes was carried out by the local computation in nodes, avoiding the regular exchange of abundant controlling information. Therefore, LLF-FR strictly restricted redundant communication in UASN and the simulation results demonstrate that LLF-FR effectively limits redundant information, improves energy efficiency, balances energy consumption, prolongs the network lifetime, reduces data conflict and data jam, and increases the packet delivery ratio compared with other protocols. It can greatly enhance the comprehensive performances of UASNs. 

## Figures and Tables

**Table 1:** Key parameters used in this paper.

Parameters	Explanation
*h*_Layer *i*_	Height of Layer *i*
*r*	Transmission range of node
Layer_Node *i*_	Layer ID of Node *i*
*d_ij_*	Distance between receiving node *j* and sending node *i*
$F_{c}\left(i\right)$	Candidate relay nodes set
*M_j_*	Metrics set for Node $j\in F_{c}\left(i\right)$
*γ_i_*	Normalized vector corresponding to each metric
H	Normalized vector matrix
Priority_*j*_	Prior of Node $j\in F_{c}\left(i\right)$
$F_{p}\left(i\right)$	Potential forwarding nodes
*α*, *μ*, *η*, *β*, *σ*	Adjustment coefficient
*E_low_*	Lowest energy limit of node to transmit data packet
*λ*	Threshold
*E_r_*	Residual energy of node
*E_o_*	Initial energy
*CoH_j_*	Coordination holding time
*C*	Ocean acoustic velocity
$\bar{C}$	Average speed of sound propagation in the ocean

**Table 2:** Related Work Summary.

Protocol	Route Metric	Feature	Address Issues	Achievements
VBF [21]	Location	“routing pipe” between sender and destination node	P3	Reduce latency and retransmissions
HH-VBF [22]	Location	“routing pipe” hop by hop	P3	Reduce VBF influence of sparse node deployment
GEDAR [16]	Location, depth	Geographic-info for candidate forwarders, depth-info for route void	P3	Avoid route void
PCR [23]	Location, PDR	Candidate forwarders under different transmission powers	P3	Save energy
ORD [10]	Location, azimuth angle, energy, PDR	Directional transmission to AUV	P3	Improve PDR, transmission latency, and energy consumption
DBR [26]	Depth	Depth based selecting relays without exchanging of location information	P2, P3	Save energy, network load; increase PDR
EEDBR [27]	Depth, energy	Depth based routing and periodically sending hello packets	P3	Prolong lifetime compared to DBR
BEAR [28]	Energy, identifying	Forwarders with higher residual; to sinks through direct or multi-hop	P3	Balance energy consumed to reduce void route; improve PDR
EnOR [29]	Energy, link reliability	Nodes ordering in routing table	P3	Balance energy consumed to reduce void route
OVAR [30]	PDR, depth	Adjacency graph adjusting forwarder count	P3	Trade-off between packet advancement and route void
EDOVE [31]	Energy, depth	Relays under neighbors energy and number, normalized depth variance	P3	Energy balancing and void avoidance
DVOR [18]	hop count	Exploit the distance vector to record smallest hop counts toward the sink	P3	Avoiding void region and long detour

**Table 3:** Simulation Parameters.

Parameter Configuration	Quantity
Transmission Range (*r*)	500 m
Packet size	200 bit
Initial energy (*E*_0_)	200 J
Omnidirectional transmission power	1 W
Directional transmission power	2 W
Receiving power (*P_r_*)	0.75 W
Data rate (*ε*)	10 kps
*γ*_0_	200 J
*γ*_1_	1000 m

## Data Availability

Not Applicable.

## Appendix A

**Theorem A1.** *According to (A1), we can obtain the difference of any two nodes CoH_j_ and CoH_k_ in*$F_{p}\left(i\right)$*, ΔCoH_jk_ satisfies*

(A1) $$|\Delta {CoH}_{jk}|\geq \frac{r}{\bar{c}}$$

*where CoH_j_ and CoH_k_, respectively, represent the CoH time of any two nodes in*$F_{p}\left(i\right)$.

**Proof of Theorem A1.** From (A2), we can obtain

(A2) $$|\Delta {CoH}_{jk}|=\left|\left(\mu {Priority}_{j}+\eta \frac{r}{C}+\beta \cdot {rand}_{j}\left(0.1,\sigma \right)\cdot \frac{r}{\bar{C}}\right)-(\mu {Priority}_{k}+\eta \frac{r}{C}+\beta \cdot {rand}_{k}\left(0.1,\sigma \right)\frac{r}{\bar{C}})\right|.$$

When multiple receiving nodes have similar ${Priority}_{j}$ and the same $\eta \frac{r}{C}$, there is

(A3) $$|\Delta {CoH}_{jk}|=\beta \cdot \left|{rand}_{j}\left(0.1,\sigma \right)-{rand}_{k}\left(0.1,\sigma \right)\right|\cdot \frac{r}{\bar{C}}$$

Then,

(A4) $$\left\{\begin{array}{c} \beta \left|{rand}_{j}-{rand}_{k}\right|=0,\;\;{\;\;\;\;\;rand}_{j}={rand}_{k}\\ \beta \left|{rand}_{j}-{rand}_{k}\right|\geq 1,\;\;\;\;\;\;{rand}_{j}\neq {rand}_{k} \end{array}\right.\left(\mathrm{when}\beta \geq 10\right)$$

Hence, we can obtain

(A5) $$\left\{\;\begin{array}{c} |\Delta {CoH}_{jk}|=0,\;{\;\;rand}_{j}\left(0.1,\sigma \right)={rand}_{k}\left(0.1,\sigma \right)\\ \;|\Delta {CoH}_{jk}|\geq \frac{r}{\bar{c}},\;\;\;{rand}_{j}\left(0.1,\sigma \right)\neq {rand}_{k}\left(0.1,\sigma \right) \end{array}\right..$$

□
